# Mapping Evidence of Pharmacy Services for COVID-19 in China

**DOI:** 10.3389/fphar.2020.555753

**Published:** 2020-10-02

**Authors:** Zhan-Miao Yi, Yang Hu, Guan-Ru Wang, Rong-Sheng Zhao

**Affiliations:** ^1^ Department of Pharmacy, Peking University Third Hospital, Beijing, China; ^2^ Institute for Drug Evaluation, Peking University Health Science Center, Beijing, China; ^3^ Therapeutic Drug Monitoring and Clinical Toxicology Center, Peking University, Beijing, China; ^4^ Department of Pharmacy Administration and Clinical Pharmacy, School of Pharmaceutical Sciences, Peking University, Beijing, China

**Keywords:** COVID-19, pharmacy service, guideline development, emergency supply, service model innovation, treatment regimens

## Abstract

**Objective:**

To explore the role of pharmacists and innovation of pharmacy interventions for COVID-19 patients in China.

**Methods:**

We comprehensively searched PubMed and Chinese databases Sinomed, China National Knowledge Infrastructure to identify articles reporting pharmacist interventions and pharmacy services for COVID-19 patients using a predefined search strategy. The search period was from inception to April 7^th^ 2020. We also manually searched the reference list of included articles and websites of important journals with special issues for COVID-19.

**Results:**

A total of 156 articles were identified by applying the search criteria. After screening, 91 articles, with the majority are literature reviews (n = 77, 84.62%) published in Chinese (n = 83, 91.2%), were included. Pharmacist interventions for COVID-19 patients most commonly related to guidelines or consensus development for the treatment of disease and practice procedure to prevent the spread of COVID-19 (n = 10, 10.98%), the supply of medicines to treat patients with severe respiratory or systematic symptoms related to COVID-19 (n = 10, 10.98%), formulating infections prevention and control strategies related to COVID-19 for pharmaceutical personnel/pharmacy staff (n = 14, 15.39%), new way of delivery pharmacy services and the implementation of new pharmacy services for COVID-19 outbreak (n = 14, 15.39%), routine pharmacy services under the restrict limit of COVID-19 outbreak (n = 39, 42.86%), and case series analysis of treatment regimens using existing, routinely collected data (n = 4, 4.40%).

**Conclusion:**

Pharmacy services have a role in the COVID-19 pandemic control, and there were many rapid changes in response to the pandemic.

## Introduction

The coronavirus disease 2019 (COVID-19) epidemic began in December 2019 in China and had spread to 210 countries and territories around the world with 4,731,458 confirmed cases as of May 19^th^ 2020 (World Health Organization, 2020a). The World Health Organization (WHO) had declared COVID-19 epidemic as a Public Health Emergency of International Concern on January 30^th^ 2020 (World Health Organization, 2020b). On March 12^th^, the WHO had declared COVID-19 epidemic a “pandemic” worldwide (World Health Organization, 2020c).

After the COVID-19 outbreak, China issued a series of emergency plans and took effective measures to control the pandemic in a timely manner. And the COVID-19 has been well-controlled in China through strenuous efforts during February 1^st^ to April 7^th^. On March 4^th^, 21 provinces in China lowered the emergency response levels. On March 19^th^ 2020, China reported no domestic new cases at the first time, indicating the ending of the first prevail peak period of COVID-19 in China. On April 7^th^, the Chinese government put forward guidelines related to actively and orderly promoting the resumption of production (Leading Group of the Central Committee on Response to COVID-19, 2020). However, as China is now facing the risk of COVID-19 rebound and most countries around the world are still at the COVID-19 pandemic lockdown, more evidence and experience are needed to successfully prevent and treat infections (Omer et al., 2020), especially for healthcare practitioners. Due to the government’s mobilization ability and Chinese medical personnel’s responsibility and courage, medical staff quickly participated in the prevention and treatment of the epidemic. For example, there were more than 42,000 medical staff supporting Hubei province in China (National Health Commission of People’s Republic of China, 2020). Meanwhile, medical resources were scientifically scheduled for COVID-19 patients at different stages. Pharmacists, as healthcare practitioner, are actively participating in the fight during the outbreak (Al-Quteimat and Amer, 2020). Pharmacists’ important role in preventing and controlling COVID-19 has been highly stressed in International Pharmaceutical Federation (FIP) guidance for pharmacists (International Pharmaceutical Federation, 2020) and the experts consensus of Chinese Pharmaceutical Association (Zhao et al., 2020a). Besides, several studies had indicated that pharmacists have a role in evidence-based therapy, monitoring and management of convalescent plasma therapy during COVID-19 pandemic in addition to the routine pharmaceutical service such as assessing patients’ previous medication history, drug allergy history, adverse drug reactions, and drug interactions (Martin et al., 2018; Song Z. W. et al., 2020).

As the forefront of the outbreak, pharmacists in China have responded rapidly to COVID-19. In the FIP guidance, community pharmacists can comfort patients, contact the health department to initiate relevant care programs, inform the public about health information about isolation, diagnosis, and treatment related procedures, and provide evidence-based information (International Pharmaceutical Federation, 2020). In China, pharmaceutical care was provided for COVID-19 patients and the public, and guidelines of clinical therapy and strategy for controlling the pandemic were promptly established and submitted to the International Pharmaceutical Federation (Zhao et al., 2020a). Moreover, many preliminary research for anti-viral treatment drugs were initiated. However, the whole picture of role of pharmacists for COVID-19 was still unclear. As China is the first country that managed the COVID-19 outbreak, the document of such experiences will be a beneficial lesson for managing the future infectious disease pandemics to come. Mapping the strategies and evidence-based strategies/measures will help further policy decision making. At the same time, the outbreak of COVID-19 has suddenly changed people’ daily life and posed challenges to the routine pharmaceutical service. As patients are under quarantine at home or in hospitals, some new ways to deliver pharmaceutical service are necessary when there is no chance of face-to-face consultation between pharmacists and patients.

Thus, we conducted a scoping review to summarize currently published articles by pharmacists, to provide information regarding content and innovation of pharmacy services for COVID-19 in China.

## Materials and Methods

The study was conducted following the methodological framework suggested by Arksey and O’Malley (2005).

### Search Strategy

We searched PubMed and two Chinese databases including Sinomed and China National Knowledge Infrastructure from inception to April 7^th^ 2020 to identify articles reporting findings related to pharmacy service for COVID-19. On April 7^th^, China announced that most areas in the country were in low risk and published the guidance for the prevention and control measures for getting back to work in different risk areas of the country (The State Council of the People’s Republic of China, 2020). The search terms included the following keywords: “COVID-19,” “severe acute respiratory syndrome coronavirus 2,” “2019-nCoV,” “SARS-CoV-2,” “Novel coronavirus,” “nCoV,” “Emerging Coronaviruses,” “new coronavirus,” “pharmacist,” “pharmacy,” “pharmaceutical care,” “pharmaceutical service,” and “medication therapy management.” Terms were translated into Chinese when searching the Chinese databases. We also manually searched the reference list of the included articles and websites of important journals as a supplementary source for relevant literature, such as Research in Social and Administrative Pharmacy, International Journal of Clinical Pharmacy, Journal of the American Pharmacists Association, American Journal of Hospital Pharmacy, Chinese Pharmaceutical Journal, China Pharmacy, Clinical Medication Journal, and Adverse Drug Reactions Journal with special issues for COVID-19. We included all the relevant publications written in English or Chinese and had no limits on the type of pharmacy service or on the country in which the service was provided. All types of publications were considered, such as original studies, reviews, editorials, commentaries, and conference preceding.

### Study Identification and Selection

Two investigators (YH and GR-W) independently screen the references of all retrieved records for potentially relevant articles, beginning with title and abstract screening in the first stage and full-text screening in the second. In the title and abstract screening stage, articles appearing to meet the inclusion criteria, or with insufficient information to make a clear judgment, were included in the full-text screening process. We obtained full texts of all these articles for the full-text screening. We included publications if they reported on pharmacy services for COVID-19 patients where either a description of the content or outcomes of pharmacy service was given. All types of publications were included. All disagreements about study selection were resolved through discussion \medicines to treat patients with severe respiratory or systematic symptoms related to COVID-19, 3) formulating infections prevention and control strategies related to COVID-19 for pharmaceutical personnel/pharmacy staff, 4) new way of delivery pharmacy services and the implementation of new pharmacy services for COVID-19 outbreak, 5) routine pharmacy services under the restrict limit of COVID-19 outbreak, and 6) case series analysis of treatment regimens using existing, routinely collected data.

### Data Extraction and Quality Assessment

Data extraction was performed by the same two investigators (YH and GR-W) using a pre-designed data collection tool. The following information was extracted from included publications: first author, publication date, type of study, content and changes of pharmacy services in response to the pandemic. Two investigators independently assessed the methodological quality of included studies. We evaluate the quality of the eligible guidelines with Appraisal of Guidelines for Research & Evaluation II (AGREE II) instrument (AGREE Next Steps Consortium, 2017). The methodological quality of eligible observational studies was evaluated with the Joanna Briggs Institute Reviewers' Manual (Moola et al., 2020).

## Results

### Study Selection and Characteristics of Included Literatures

**Figure 1:**
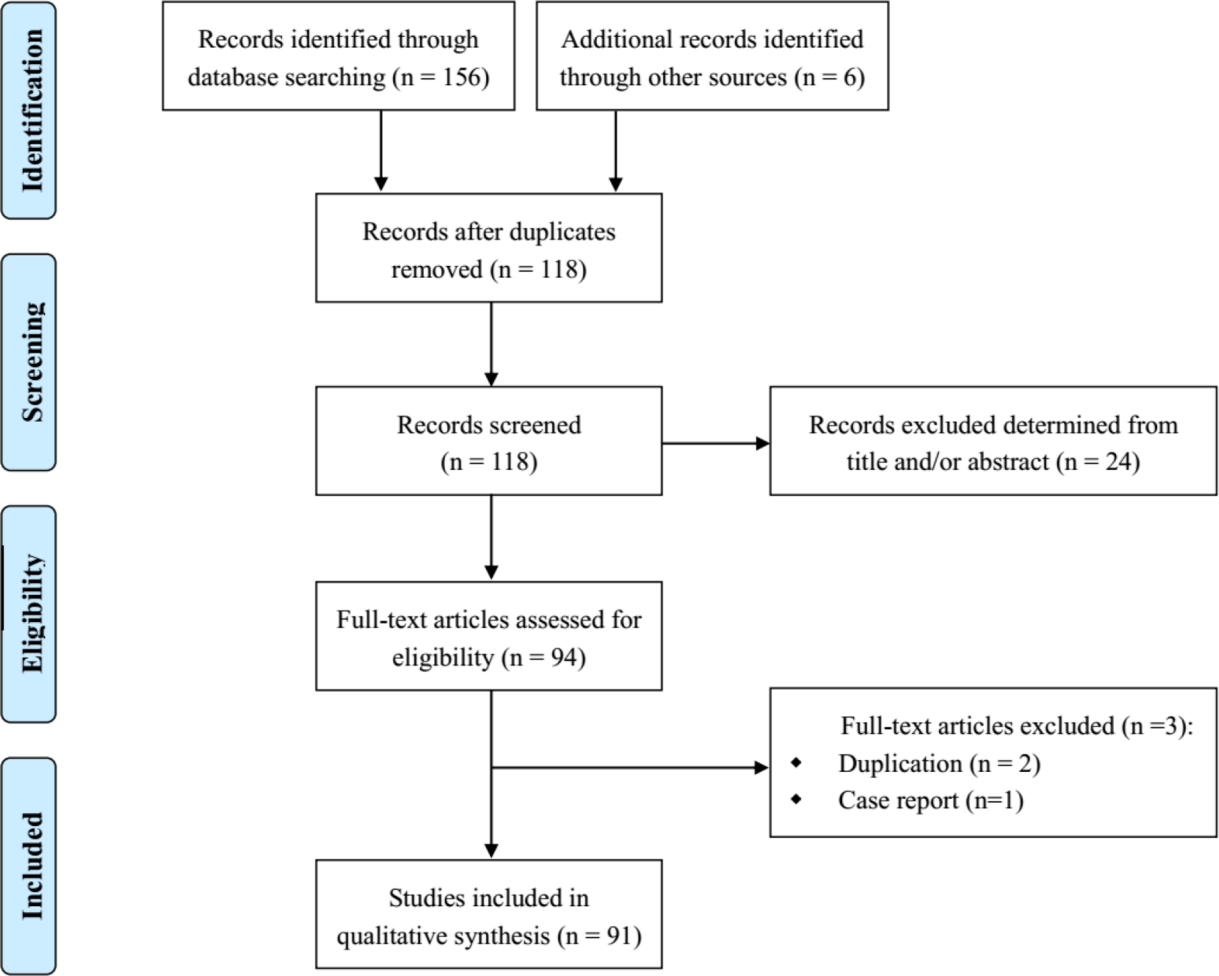
Flow diagram for literature search and study selection.

The initial search identified 156 relevant records, with a further two additional records identified through other sources (journal websites). Of these, 68 were excluded after duplicates were removed and title/abstract screening, leaving 94 papers eligible for full-text review. Ninety one articles that met the inclusion criteria were finally included (Bian et al., 2020a; Bian et al., 2020b; Cao et al., 2020; Che et al., 2020; Chen et al., 2020a; Chen et al., 2020b; Chen F. et al., 2020; Chen J. Z. et al., 2020; Chen L. F. et al., 2020; Chen L. P. et al., 2020; Chen M. et al., 2020; Chen Y. W. et al., 2020; Dong et al., 2020; Du et al., 2020; Duan et al., 2020; Gao et al., 2020; Gong et al., 2020a; Gong et al., 2020b; Guo et al., 2020; Gu et al., 2020; Han et al., 2020; He L. L. et al., 2020; He S. Z. et al., 2020; Hu C. J. et al., 2020; Hu Z. Q. et al., 2020; Huang et al., 2020a; Huang et al., 2020b; Huang L. F. et al., 2020; Jin et al., 2020; Li G. H. et al., 2020; Li Q. C. et al., 2020; Lin and Zhang, 2020; Liu and Yan, 2020; Liu X. X. et al., 2020; Liu et al., 2020a; Jin et al., 2020; Li H. et al., 2020; Liu et al., 2020b; Liu X. L. et al., 2020; Liu Y. et al., 2020; Meng et al., 2020; Peng et al., 2020; Rao et al., 2020; Shao et al., 2020; Shen et al., 2020; Song B. et al., 2020; Song Z. W. et al., 2020; Tan et al., 2020; Therapeutic Drug Monitoring Pharmacists Branch of Chinese Pharmacists Association et al., 2020; Ung, 2020; Wang, 2020; Wang J. W. et al., 2020; Wang N. N. et al., 2020; Wang R. R. et al., 2020; Wang Y. et al., 2020; Wei et al., 2020; Xie et al., 2020; Xiong et al., 2020a; Xiong et al., 2020b; Xu X. H. et al., 2020; Xu Y. X. et al., 2020; Yang Y. Y. et al., 2020; Yang et al., 2020a; Yang et al., 2020b; Yan J. L. et al., 2020; Yan Y. Y. et al., 2020; Yang M. Y. et al., 2020; Yang Y. et al., 2020; Yang C. et al., 2020; Yang L. et al., 2020; Yang L. et al., 2020; Yang Y. H. et al., 2020; Yi et al., 2020; Yin et al., 2020; Ying et al., 2020; Yuan et al., 2020; Zeng et al., 2020; Zhang D. et al., 2020; Zhang J. H. et al., 2020; Zhang L. et al., 2020; Zhang X. Q. et al., 2020; Zhang Y. L. et al., 2020; Zhang Z. H. et al., 2020; Zheng et al., 2020; Zhao et al., 2020a; Zhao et al., 2020b; Zhao et al., 2020c; Zhou H. et al., 2020; Zhou P. X. et al., 2020; Zhou W. et al., 2020; Zhu S. Y. et al., 2020; Zhu Y. G. et al., 2020). **Figure 1** provides details of the reasons for excluding articles from this review.

### Research Domains, Publication Dates, and Methodological Characteristics

**Figure 2:**
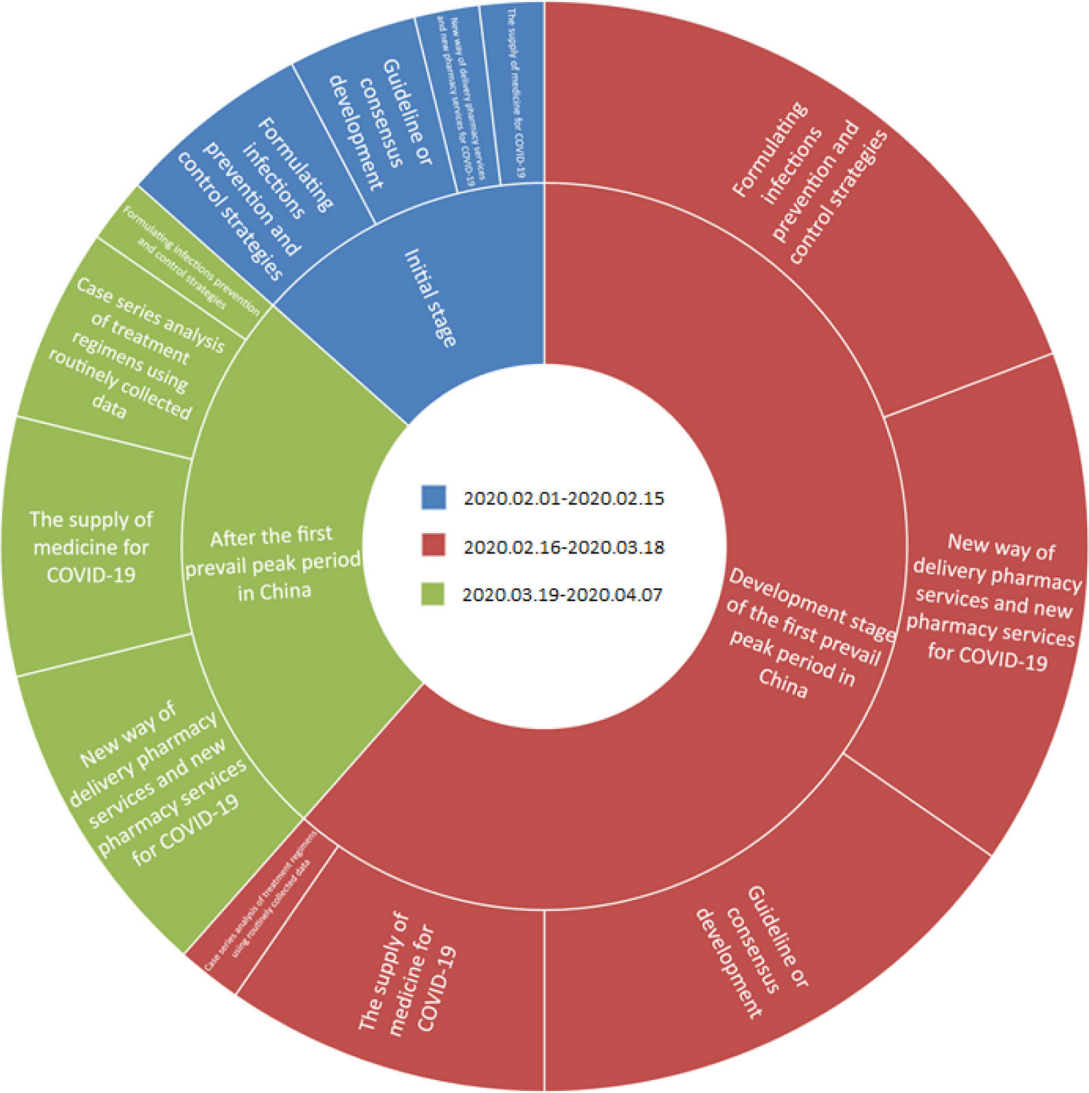
Contents and publication dates of included articles.

Most of the publications (n = 83, 91.2%) (Bian et al., 2020a; Bian et al., 2020b; Cao et al., 2020; Che et al., 2020; Chen et al., 2020a; Chen et al., 2020b; Chen F. et al., 2020; Chen J. Z. et al., 2020; Chen L. F. et al., 2020; Chen L. P. et al., 2020; Chen M. et al., 2020; Chen Y. W. et al., 2020; Dong et al., 2020; Du et al., 2020; Duan et al., 2020; Gao et al., 2020; Gong et al., 2020a; Gong et al., 2020b; Gu et al., 2020; Guo et al., 2020; Han et al., 2020; He L. L. et al., 2020; He S. Z. et al., 2020; Hu C. J. et al., 2020; Hu Z. Q. et al., 2020; Huang et al., 2020a; Huang et al., 2020b; Huang L. F. et al., 2020; Jin et al., 2020; Li G. H. et al., 2020; Li Q. C. et al., 2020; Lin and Zhang, 2020; Liu and Yan, 2020; Liu X. X. et al., 2020; Liu et al., 2020b; Liu X. L. et al., 2020; Liu Y. et al., 2020; Peng et al., 2020; Rao et al., 2020; Shao et al., 2020; Shen et al., 2020; Song B. et al., 2020; Tan et al., 2020; Therapeutic Drug Monitoring Pharmacists Branch of Chinese Pharmacists Association et al., 2020; Wei et al., 2020; Wang, 2020; Wang J. W. et al., 2020; Wang N. N. et al., 2020; Wang R. R. et al., 2020; Wang Y. et al., 2020; Xie et al., 2020; Xiong et al., 2020a; Xiong et al., 2020b; Xu X. H. et al., 2020; Xu Y. X. et al., 2020; Yan J. L. et al., 2020; Yan Y. Y. et al., 2020; Yang et al., 2020b; Yang L. et al., 2020; Yang Y. H. et al., 2020; Yang Y. Y. et al., 2020;Yang L. et al., 2020; Yang et al., 2020a; ; Yang M. Y. et al., 2020; Yang Y. et al., 2020; Yang C. et al., 2020; Yi et al., 2020; Yin et al., 2020; Yuan et al., 2020; Zeng et al., 2020; Zhang D. et al., 2020; Zhang J. H. et al., 2020; Zhang L. et al., 2020; Zhang X. Q. et al., 2020; Zhang Y. L. et al., 2020; Zhang Z. H. et al., 2020; Zhao et al., 2020a; Zhao et al., 2020b; Zhao et al., 2020c; Zhou H. et al., 2020; Zhou P. X. et al., 2020; Zhu S. Y. et al., 2020; Zhu Y. G. et al., 2020) are in Chinese and few (n = 8, 8.8%) are in English (Song Z. W. et al., 2020; Zhou W. et al., 2020; Zheng et al., 2020; Liu et al., 2020a; Meng et al., 2020; Li H. et al., 2020; Ying et al., 2020; Ung, 2020). The publications mainly focused on routine pharmacy services under the restrict limit of COVID-19 outbreak (42.86%) (**Table 1**). Most of articles were published in March 2020, with increased articles on retrospective analysis of treatment regimens (**Figure 2**).

**Table 1:** Research domains of included articles.

Research domains	English literature		Chinese literature		Total	
	n	%	n	%	n	%
Guideline or consensus development	0	0	10	10.98	10	10.98
The supply of medicines for COVID-19	1	1.10	9	9.88	10	10.98
Formulating infections prevention and control strategies related to COVID-19	1	1.10	13	14.29	14	15.39
New way of delivery pharmacy services and new pharmacy services for COVID-19	3	3.30	11	12.09	14	15.39
Routine pharmacy services under the restrict limit of COVID-19 outbreak	3	3.30	36	39.56	39	42.86
Case series analysis of treatment regimens	0	0	4	4.40	4	4.40

We also analyzed the methodological characteristics of the publications in the final sample. The majority of included articles are literature review (n = 77, 84.62%), and others were guidelines/consensus (n = 10, 10.99%) and original research (n = 4, 4.40%).

### Geographic Distributions of Pharmacy Service Literatures

**Figure 3:**
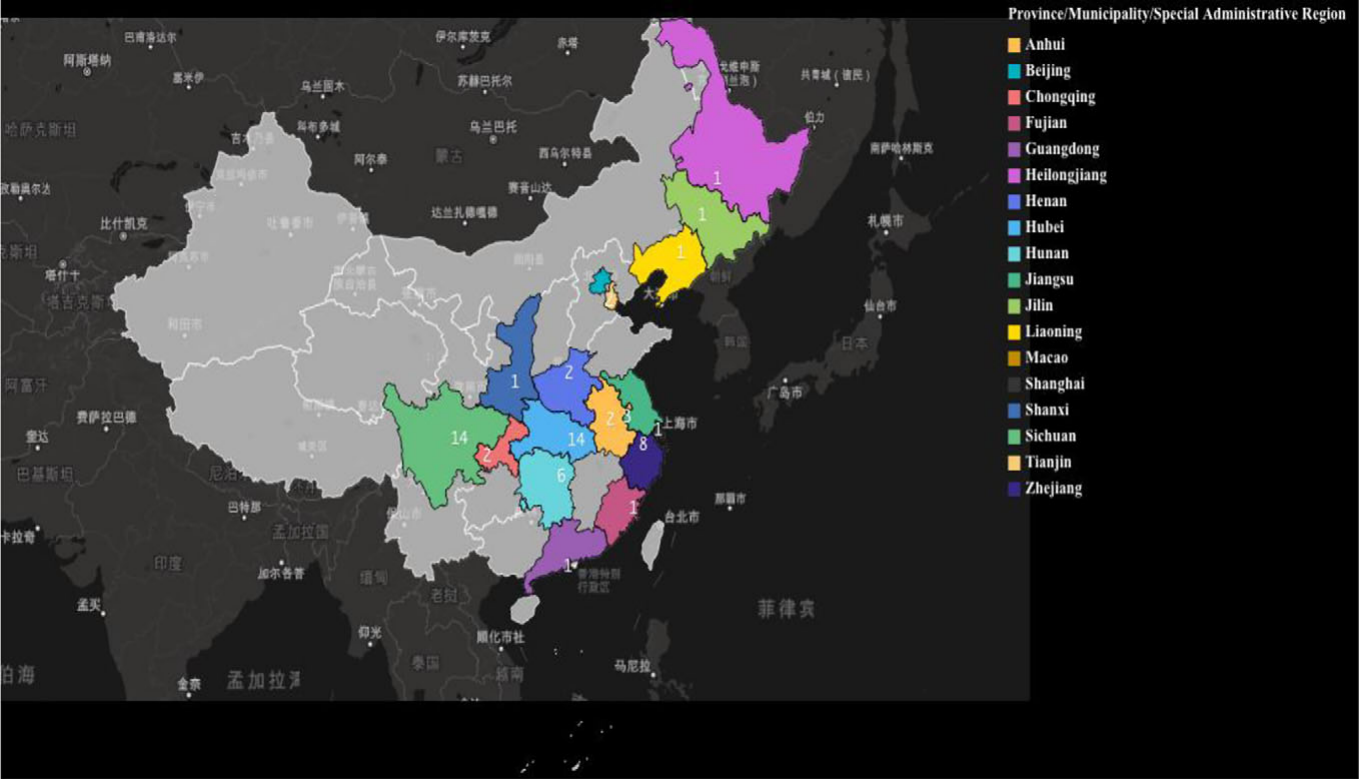
Geographic distributions of pharmacy service articles for COVID-19 in China.

Pharmacists from 18 provinces of China published Chinese and English articles on pharmacy service, with pharmacists from Beijing (n = 26, 28.57%) (Dong et al., 2020; Guo et al., 2020; International Pharmaceutical Federation, 2020; Jin et al., 2020; Li H. et al., 2020; Liu and Yan, 2020; Lin and Zhang, 2020; Song Z. W. et al., 2020; Therapeutic Drug Monitoring Pharmacists Branch of Chinese Pharmacists Association et al., 2020; Xu X. H. et al., 2020; Yan J. L. et al., 2020; Yan Y. Y. et al., 2020; Yang L. et al., 2020; Yang M. Y. et al., 2020; Yang Y. H. et al., 2020; Yi et al., 2020; Yin et al., 2020; Zhang D. et al., 2020; Zhang L. et al., 2020; Zhang Y. L. et al., 2020; Zhang Z. H. et al., 2020; Zhao et al., 2020b; Zhao et al., 2020c; Zheng et al., 2020; Zhou P. X. et al., 2020; Zhou W. et al., 2020), Sichuan (n = 14, 15.38%) (Bian et al., 2020a; Bian et al., 2020b; Chen M. et al., 2020; Du et al., 2020; Gao et al., 2020; Han et al., 2020; He L. L. et al., 2020; Hu C. J. et al., 2020; Hu Z. Q. et al., 2020; Li Q. C. et al., 2020; Liu X. X. et al., 2020; Xiong et al., 2020a; Xiong et al., 2020b; Yang Y. et al., 2020), and Hubei (n = 14, 15.38%) (Chen et al., 2020a; Chen J. Z. et al., 2020; Gong et al., 2020a; Gong et al., 2020b; Gu et al., 2020; Huang et al., 2020a; Huang et al., 2020b; Li G. H. et al., 2020; Liu X. L. et al., 2020; Liu Y. et al., 2020; Yang et al., 2020a; Yang et al., 2020b; Zhou H. et al., 2020) published most of the articles (**Figure 3**). Statistics on the geographic areas of pharmacy workers who published papers in China can explain to a certain extent the differences in the level of medical technology in different regions of China and the speed with which pharmacy workers respond to epidemics.

### Contents of Pharmacy Services

**Figure 4:**
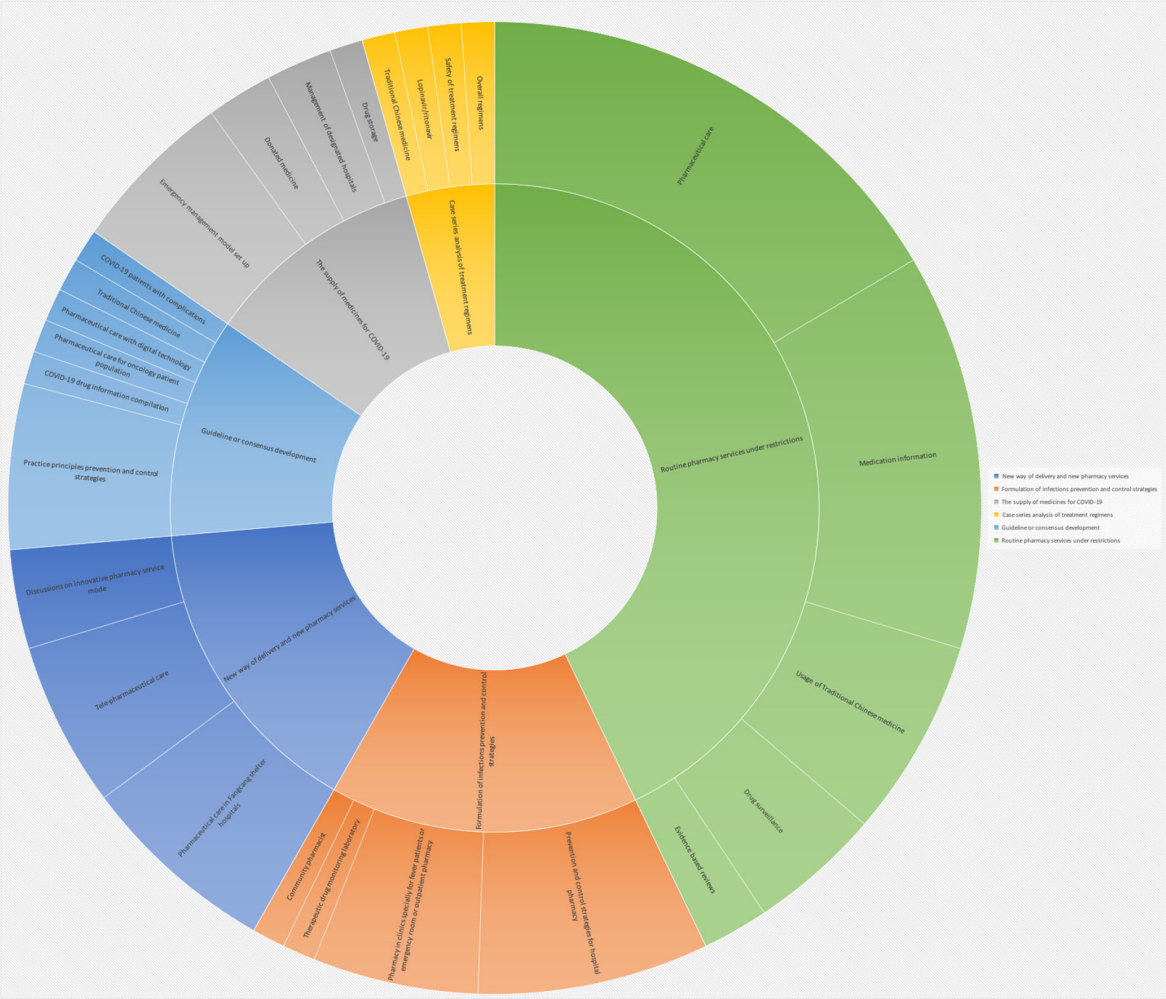
Sunburst plot of pharmacy service articles for COVID-19 in China. *Some articles focused on more than one content in the subgroups.

For contents of included 91 articles, 39 articles (42.86%) were related to routine pharmacy services under the restrict limit of COVID-19 outbreak (Bian et al., 2020a; Cao et al., 2020; Che et al., 2020; Chen et al., 2020a; Chen L. P. et al., 2020; Chen M. et al., 2020; Chen Y. W. et al., 2020; Du et al., 2020; Duan et al., 2020; Guo et al., 2020; Han et al., 2020; Hu Z. Q. et al., 2020; Lin and Zhang, 2020; Li Q. C. et al., 2020; Liu and Yan, 2020; Liu X. X. et al., 2020; Peng et al., 2020; Shao et al., 2020; Song B. et al., 2020; Song Z. W. et al., 2020; Wang, 2020; Wang Y. et al., 2020; Wang R. R. et al., 2020; Xiong et al., 2020a; Xiong et al., 2020b; Yang C. et al., 2020; Yang et al., 2020a; Yang et al., 2020b; Yan J. L. et al., 2020; Yang L. et al., 2020; Yang M. Y. et al., 2020; Yi et al., 2020; Zhang D. et al., 2020; Zhang J. H. et al., 2020; Zhang Y. L. et al., 2020; Zhang Z. H. et al., 2020; Zheng et al., 2020; Zhou W. et al., 2020; Zhu S. Y. et al., 2020), 14 articles (15.39%) focused on new way of delivery pharmacy services to reduce face-to-face contact using innovative technologies and the implementation of new pharmacy services for COVID-19 outbreak (16.28%) (Liu et al., 2020a; Meng et al., 2020; Huang L. F. et al., 2020; Yang Y. Y. et al., 2020; Zeng et al., 2020; Bian et al., 2020b; Gao et al., 2020; He L. L. et al., 2020; Gu et al., 2020; Li H. et al., 2020; Gong et al., 2020a; Gong et al., 2020b; Chen F. et al., 2020; Yang Y. et al., 2020), 14 articles (15.39%) focused on the formulation of infections prevention and control strategies for pharmacy workforce (Chen J. Z. et al., 2020; Chen L. F. et al., 2020; Shen et al., 2020; Tan et al., 2020; Ung, 2020; Wang N. N. et al., 2020; Wei et al., 2020; Yang L. et al., 2020; Yan Y. Y. et al., 2020; Yuan et al., 2020; Zhang X. Q. et al., 2020; Zhou H. et al., 2020; Zhou P. X. et al., 2020), 10 articles (10.99%) were guidelines or expert consensus (International Pharmaceutical Federation, 2020; Jin et al., 2020; Li G. H. et al., 2020; Therapeutic Drug Monitoring Pharmacists Branch of Chinese Pharmacists Association et al., 2020; Wang J. W. et al., 2020; Yang Y. H. et al., 2020; Zhang L. et al., 2020; Zhao et al., 2020b; Zhao et al., 2020c; Zhu Y. G. et al., 2020), 10 articles (10.99%) introduced the supply of medicines for COVID-19 during pandemic in China (Dong et al., 2020; He S. Z. et al., 2020; Hu C. J. et al., 2020; Liu et al., 2020b; Liu X. L. et al., 2020; Liu Y. et al., 2020; Rao et al., 2020; Xie et al., 2020; Xu X. H. et al., 2020; Ying et al., 2020), and 4 articles (4.40%) were case series analysis of the effectiveness and safety of treatment regimens for COVID-19 (Chen et al., 2020b; Huang et al., 2020a; Huang et al., 2020b; Xu Y. X. et al., 2020) (**Figure 4**).

### Guideline or Consensus Development

These articles introduced work guidance on drug management and usage in special periods and the plan and response strategy by Chinese pharmacy personnel for COVID-19 pandemic situation from a macro perspective. The domains that scored better were: “scope and purpose” (all included guidelines/consensus exceeding the cut-off of 70%) and “clarity and presentation” (5 of 10 included guidelines/consensus exceeding the cut-off of 70%). Those that scored worse were “stakeholder involvement” and “rigour of development” (none of the included guidelines/consensus exceeding the cut-off of 70%). Two of the included guidelines/consensus had three domains exceeded the cut-off of 70% (International Pharmaceutical Federation, 2020; Zhao et al., 2020b), while others had only one or two domains exceeded the cut-off of 70%. Topics of guidelines or expert consensus included practice principles prevention and control strategies for hospital or retail pharmacy (n = 5) (International Pharmaceutical Federation, 2020; Wang J. W. et al., 2020; Yang Y. H. et al., 2020; Zhao et al., 2020b; Zhao et al., 2020c), COVID-19 drug information compilation (n = 1) (Zhu Y. G. et al., 2020), traditional Chinese medicine (n = 1) (Jin et al., 2020), pharmaceutical care for oncology patient population (n = 1) (Li G. H. et al., 2020) COVID-19 patients with complications (n = 1) (Therapeutic Drug Monitoring Pharmacists Branch of Chinese Pharmacists Association et al., 2020), and pharmaceutical care with digital technology (n = 1) (Zhang L. et al., 2020). For example, the “Novel Coronavirus Infection: Expert Consensus on Guidance and Prevention Strategies for Hospital Pharmacists and the Pharmacy Workforce” formulated by Peking University Third Hospital has carried out analysis and summary of relevant data in the process of epidemic prevention and control (Zhu Y. G. et al., 2020). The consensus of the expert was published on February 7^th^, when there were relatively limited information and response measures for COVID-19. The expert consensus summarized information needed to continue pharmacy services during the epidemic, and provided reference and experience for Chinese pharmacy personnel in a timely manner.

### The Supply of Medicines for COVID-19

Topics of these articles covered the emergency management model set up (n = 5) (Dong et al., 2020; Liu et al., 2020b; Rao et al., 2020; Xie et al., 2020; Ying et al., 2020) and the management of designated hospitals (n = 2) (Liu X. L. et al., 2020; Liu Y. et al., 2020), donated medicines (n = 2) (He S. Z. et al., 2020; Xu X. H. et al., 2020), and drug storage (n = 1) (Hu C. J. et al., 2020) in hospital pharmacy. For example, the study “Pharmaceutical emergency guarantee difficulties and countermeasures for the prevention and control of outbreak of COVID-19” by Xiangya Hospital published in February summarized four main issues on the supply of medicine for epidemic prevention and control (Liu et al., 2020b).

### Formulating Infections Prevention and Control Strategies

These articles analyze and summarize training of pharmacists during the epidemic, methods of wearing personal protective equipment, disinfection measures, psychological counseling, etc, aiming to reduce risk of infection of pharmacy staff and strengthen the health management of pharmacy staff. Topics covered hospital pharmacists (n = 7) (Chen J. Z. et al., 2020; Tan et al., 2020; Yang L. et al., 2020; Yan Y. Y. et al., 2020; Yin et al., 2020; Zhang X. Q. et al., 2020; Zhou P. X. et al., 2020), community pharmacists (n = 1) (Ung, 2020), pharmacy in clinics specially for fever patients or emergency room or outpatient pharmacy (n = 5) (Chen L. F. et al., 2020; Shen et al., 2020; Wang N. N. et al., 2020; Yuan et al., 2020; Zhou P. X. et al., 2020), therapeutic drug monitoring laboratory (n = 1) (Zhou H. et al., 2020). Workforce in the drug monitoring laboratory also has a high risk of infection. One study by pharmacists in Wuhan formulated detailed prevention and control strategies for pharmacists conducting therapeutic drug monitoring (TDM) and provided experience for the development of personal protection, prevention, and control strategies for pharmacists of the same position (Zhou H. et al., 2020). During the epidemic period, pharmacists conducting TDM should handle a large number of blood samples every day, thus the occupational exposure protection and the management of laboratory biological safety were very important.

### New Way of Delivery Pharmacy Services and New Pharmacy Services for COVID-19

Topics of these articles covered pharmaceutical care in Fangcang sheltered hospitals (n = 6) (Chen F. et al., 2020; Gong et al., 2020a; Gong et al., 2020b; Gu et al., 2020; Meng et al., 2020; Yang Y. et al., 2020), telepharmaceutical care (n = 5) (Gao et al., 2020; He L. L. et al., 2020; Li H. et al., 2020; Yang Y. Y. et al., 2020; Zeng et al., 2020), and other articles on innovative pharmacy service models (n = 3) (Liu et al., 2020a; Huang L. F. et al., 2020; Bian et al., 2020b), such as applying models and practical methods to ensure the quality and accessibility of pharmacy services. In the study “The practice and discussion of the online pharmaceutical service mode in square cabin hospital” by pharmacists in the frontline of the Fangcang shelter hospitals explored online methods such as WeChat to provide pharmacy services such as medication reconciliation and patient education (Gong et al., 2020b). The shelter hospital, a new public health initiative in a special period, is built by a number of movable modules and has many functions such as emergency treatment and clinical testing. As a necessary functional module for the shelter hospital, the shelter pharmacy played an important role in ensuring the supply of medicines for COVID-19 patients. This article explored new way of delivering pharmacy services for different patients in shelter hospital, and showed the responsibility and creativity of Chinese pharmacists during the pandemic.

### Routine Pharmacy Services Under the Restrict Limit of COVID-19 Outbreak

These articles mainly introduced the key points of patient monitoring during the epidemic, including off-label use, safety use of drugs in clinical trials, and special populations. Topics covered medication information (n = 12) (Bian et al., 2020a; Cao et al., 2020; Che et al., 2020; Chen Y. W. et al., 2020; Du et al., 2020; Liu and Yan, 2020; Liu X. X. et al., 2020; Yang M. Y. et al., 2020; Yan J. L. et al., 2020; Zhang J. H. et al., 2020; Zhang Z. H. et al., 2020; Zhou W. et al., 2020), usage of traditional Chinese medicine (n = 6) (Chen et al., 2020a; Duan et al., 2020; Lin and Zhang, 2020; Yang C. et al., 2020; Wang, 2020; Zhang Y. L. et al., 2020), pharmaceutical care (n = 15) (Guo et al., 2020; Hu Z. Q. et al., 2020; Li Q. C. et al., 2020; Shao et al., 2020; Song B. et al., 2020; Song Z. W. et al., 2020; Peng et al., 2020; Wang R. R. et al., 2020; Wang Y. et al., 2020; Xiong et al., 2020a; Xiong et al., 2020b; Yang et al., 2020a; Yang et al., 2020b; Yang L. et al., 2020; Zheng et al., 2020), evidence based review for interferon and lopinavir/ritonavir (n = 2) (Chen M. et al., 2020; Yi et al., 2020), and drug surveillance (n = 4) (Chen L. P. et al., 2020; Han et al., 2020; Zhang D. et al., 2020; Zhu S. Y. et al., 2020).

### Case Series Analysis of Treatment Regimens Using Routinely Collected Data

In general, the quality of included case series was good [two scored 16 (Chen et al., 2020b; Xu Y. X. et al., 2020) and another two scored 18 (Huang et al., 2020a; Huang et al., 2020b) of 20 points]. Case-series study by Chen et al. indicated that all patients of 131 cases with COVID-19 treated with traditional Chinese medicine (TCM) Ganlu Xiaodu Decoction and other medicine were cured and discharged (Chen et al., 2020b) and indicated a certain effectiveness for COVID-19. However, the rationality and safety of such therapeutic schedule still need further verification. Another case-series study of 71 patients with COVID-19 by Huang et al. indicated that the combination therapy of α-interferon, arbidol, high-dose vitamin C, and TCM were effective (Huang et al., 2020a). The remaining two articles retrospectively reviewed the safety of therapeutic schedule for COVID-19 (Huang et al., 2020b; Xu Y. X. et al., 2020). Xu et al. observed the adverse drug reactions of lopinavir/ritonavir for severe COVID-19 infection in seven patients (Xu Y. X. et al., 2020) and indicated that lopinavir/ritonavir could cause serious adverse drug reactions and that safety monitoring was needed. Huang et al. conducted a case-series study of 71 confirmed case of COVID-19 and indicated that inappropriate application of antibiotics for COVID-19 should be avoided and multi-drug combinations could increase the risk of adverse drug reactions (Huang et al., 2020b).

## Discussion

The first evidence map with a total of 91 articles provided a comprehensive summary of articles of pharmacy services in the early stage in response to the COVID-19 in China. Majority are providing routine pharmaceutical care and medication information, especially for special population under the restrict limit of COVID-19 outbreak, prevention and control strategies for hospital pharmacy, clinics specially for fever patients or emergency room, and emergency management model set up. Pharmaceutical care in Fangcang shelter hospitals is new pharmacy service and tele-pharmaceutical care is the main new way to deliver pharmacy service during the pandemic. The included articles were mainly literature reviews in Chinese from three provinces published during March, with Beijing and Sichuan having strong academic capabilities and Hubei the epic center of COVID-19 (Yan et al., 2012).

As the earliest country to fight against COVID-19, the published articles provided important experience of pharmacists in China at the frontline. In the initial stage of the pandemic (February 1^st^ 2020 to February 15^th^ 2020), pharmacists acted quickly mainly to formulate infection prevention and control strategies and to give suggestions on providing routine pharmaceutical care in response to the outbreak. The emergency management models of supplying of medicine for COVID-19 began to be set up at the same time. In the development stage of the first prevail peak period of the pandemic in China (February 16^th^ 2020 to March 18^th^ 2020), pharmacists mainly involved in patient management, formulating guidelines and consensus, and updating infections prevention and control strategies as the pandemic situation changes. Meanwhile, pharmacists were actively innovating the new ways of delivery pharmacy services and new pharmacy services for COVID-19. From March 19^th^ 2020 to April 7^th^ 2020, pharmacists consolidated evidence on the effectiveness of anti-epidemic, resumed medical work properly, and summarized the work experience in the pandemic to better respond to public health emergencies in the future.

However, our study has several limitations. Firstly, the current articles mainly focused on strategies at the beginning and there are limited evidence on the effectiveness and safety of medication for COVID-19 (National Institute for Health and Care Excellence, 2020). Secondly, there are limited, including original, research of pharmacy interventions. As articles on retrospective analysis of treatment regimens increased, more original research on the effectiveness and safety of current treatments is needed, especially for off-label use, outcome evaluation, and real-world data analysis. Thirdly, many high-quality international journals may take longer to publish manuscripts on COVID-19, and thus it is likely to miss out some relevant articles.

Comparing with another scoping review on epidemiology, causes, clinical manifestation and diagnosis, prevention, and control of COVID-19, which indicated 89.2% articles were published in English (Adhikari et al., 2020), pharmacy services articles were mainly published in Chinese, perhaps to facilitate domestic communications among pharmacists during the early stage of the outbreak. Thus an evidence mapping can provide information and experience for pharmacists around the world during global pandemic. A health belief model for understanding the reasons of individuals may or may not act facing a threat to personal or community health was suggested for community pharmacists (Carico et al., 2020). This evidence mapping study can provide information to design a complex intervention following the guidance produced by the Medical Research Council (2019).

## Conclusions

Pharmacy services have a role in the COVID-19 pandemic control and there were many rapid changes in response to the pandemic. However, more original research, especially outcome evaluation and real-world data analysis, is needed to provide valid and reliable ways for curbing this pandemic

## Data Availability Statement

The raw data supporting the conclusions of this article will be made available by the authors, without undue reservation, to any qualified researcher.

## Ethics Statement

Ethical review and approval was not required for the study on human participants in accordance with the local legislation and institutional requirements. Written informed consent for participation was not required for this study in accordance with the national legislation and the institutional requirements.

## Author Contributions

Z-MY conceived this review. YH and G-RW identified reports of trials and extracted data. Z-MY, YH and G-RW did all statistical analyses, checked for statistical inconsistency, and interpreted data. R-SZ contributed to data interpretation. Z-MY drafted the report and all other authors (YH, G-RW, and R-SZ) critically reviewed the article. All authors contributed to the article and approved the submitted version.

## Conflict of Interest

The authors declare that the research was conducted in the absence of any commercial or financial relationships that could be construed as a potential conflict of interest.
